# A brief reference to AI-driven audible reality (AuRa) in open world: potential, applications, and evaluation

**DOI:** 10.3389/frai.2024.1424371

**Published:** 2024-10-25

**Authors:** Ömer Ates, Garima Pandey, Athanasios Gousiopoulos, Theodoros G. Soldatos

**Affiliations:** ^1^School of Information, Media, and Design, SRH Hochschule Heidelberg, SRH University of Applied Science, Heidelberg, Germany; ^2^Department of Library, Archives and Information Systems, School of Social Sciences, International Hellenic University, Thessaloniki, Greece; ^3^Department of Accounting and Information Systems, School of Economics and Business Administration, International Hellenic University, Thessaloniki, Greece

**Keywords:** digital health, public health, object recognition, text to speech, visual aid companion, vision impairment, real world decision support, biomedicine and healthcare informatics

## Abstract

Recent developments on artificial intelligence (AI) and machine learning (ML) techniques are expected to have significant impact on public health in several ways. Indeed, modern AI/ML methods have been applied on multiple occasions on topics ranging from drug discovery and disease diagnostics to personalized medicine, medical imaging, and healthcare operations. While such developments may improve several quality-of-life aspects (such as access to health services and education), it is important considering that some individuals may face more challenges, particularly in extreme or emergency situations. In this work, we focus on utilizing AI/ML components to support scenarios when visual impairment or other limitations hinder the ability to interpret the world in this way. Specifically, we discuss the potential and the feasibility of automatically transferring key visual information into audio communication, in different languages and in real-time—a setting which we name ‘*au*dible *r*e*a*lity’ (AuRa). We provide a short guide to practical options currently available for implementing similar solutions and summarize key aspects for evaluating their scope. Finally, we discuss diverse settings and functionalities that AuRA applications could have in terms of broader impact, from a social and public health context, and invite the community to further such digital solutions and perspectives soon.

## 1 Introduction

Recent artificial intelligence (AI) and machine learning (ML) developments are expected to significantly impact public health. Applications range from drug discovery and disease diagnostics to personalized medicine, healthcare operations, and evidence-based real-world (RW) analytics (e.g., Bohr and Memarzadeh, 2020; Vamathevan et al., 2019; Schneider et al., 2020; Lee and Yoon, 2021; Goecks et al., 2020; Elemento et al., 2021; Soldatos et al., 2022; Soldatos et al., 2019; Liu et al., 2023; Brock et al., 2022; Brock et al., 2023; Mullowney et al., 2023; Wang et al., 2024; Olawade et al., 2023). While AI/ML advancements may increase access to health services and improve the quality-of-life for many, challenges may persist for some, particularly in emergency situations (e.g., Kuriakose et al., 2023; Chenais et al., 2023; Grant et al., 2020; Ahmed et al., 2023). One such group that can benefit from modern AI solutions on computer vision and text-to-speech (TTS) technologies is visually impaired individuals (e.g., Kuriakose et al., 2023).

Computer vision is concerned with the development of algorithms and techniques that allow machines to analyze, process, and interpret digital images and videos. A small list of tasks pertaining image/video processing are listed in Table 1 (upper part). For example, in recent years object detection/recognition (ODR) is increasingly leveraged to assist in visually navigating environments (e.g., in autonomous vehicles; Tapu et al., 2017; Masmoudi et al., 2019; Malligere Shivanna and Guo, 2023). However, relying solely on visual input can be limiting for individuals with significant visual impairments, as it may not provide a comprehensive understanding of their surroundings. To address this challenge, researchers have explored the use of alternative modalities, such as of audio (e.g., Park and Fine, 2023; Maimon et al., 2023; Shvadron et al., 2023; Gamage et al., 2023; Neugebauer et al., 2020), which can enhance ODR and improve the experience and independence of visually impaired persons.

**Table 1:** Common image/video processing tasks and popular of text-to-speech (TTS) applications.

Common image/video processing tasks	
Task	Description of goal
Object detection (or object recognition)	To determine what objects are present. Given an input image, class labels and probabilities of the objects likely contained in that image are extracted (see, Figure 1). Boxes determining the position of these objects in the image may also be extracted.
Image classification	To assign a label or class to an entire image. Given an input image, a prediction about which class the image belongs to is returned.
Segmentation	To assign labels to the pixels of an image. Specifically, semantic segmentation is the process of classifying every pixel in an image into a specific class or category, instance segmentation is the process of detecting individual objects in an image (while also distinguishing between objects of the same class), whereas panoptic segmentation is used to assign a unique label to every pixel in an image (with pixels that do not belong to any object instance being assigned to ‘background’).
Text information	To detect regions in an image which contain text. The text mentioned in these regions may be processed further to extract the mentioned information (e.g., speed limit from a sign board).
Action recognition	To recognize which (human) actions or activities are performed, in which sequence of frames, in which time interval, and in which location in the scene the acting person(s) is/are. Spatio-temporal action detection is focused on determining the regions and times of an action class, whereas skeleton-based action detection is concerned with capturing (human) actions as represented by motions of skeleton.
Object tracking	To trace the location of objects in images or video frames. Single object tracking is concerned with a single object throughout a video sequence, while multiple object tracking involves tracking multiple objects simultaneously in the same video sequence. In single object tracking, the goal is to maintain the identity of the object being tracked over time, whereas in multiple object tracking, the goal is to simultaneously track and maintain the identities of multiple objects in the same scene. In a sense the goal is to trace the instances of object(s) appearing across different frames, even when undergoing changes in appearance, orientation, and movement.
Popular applications of TTS technology	
Application	Description of scope
Voice assistants	Software to execute tasks via voice command. Some of the most popular virtual voice assistants include Apple’s Siri (Siri, 2024), Amazon’s Alexa (Amazon Alexa Voice AI | Alexa Developer Official Site, 2024), Microsoft’s Cortana (Cortana, 2024), and Google’s Assistant (Google Assistant, 2024). Smart TTS technology ensures smooth and efficient communication between users and application, providing a more natural and human-like interaction between them and the used devices.
Customer service automation	Interactive voice response systems may combine pre-recorded messages or TTS technology to engage with customers, allowing them to provide and access information without employing a human agent.
Education and learning	TTS technology can help education and learning in various ways. It can make reading and understanding text easier for individuals who struggle with traditional reading methods, such as those with dyslexia or visual impairments. TTS can also help learners with limited reading proficiency to access digital content, including textbooks and online resources, as well as improve their pronunciation and fluency in a foreign language. In addition, TTS can provide a multi-sensory learning experience, which can be helpful for learners with different learning styles.
Audio books	Converting written text (such as books, news, magazine articles or websites) into spoken words can be particularly helpful for those with visual impairments or reading difficulties.
Navigation tools	Provision of instructions and directions in voice to users (e.g., to a driver or to a pedestrian) or reading out the names of streets and landmarks makes it easier to navigate without having to look at a map or screen. Additionally, TTS can be used to alert the user of upcoming turns, changes in direction, and other important information related to navigation.
Travel and tourism	TTS technology can help travelers to cope with real-time information (e.g., travel announcements in airports and train stations) by providing audio narratives translated in the language of their choice.

TTS technology is one such modality that can provide auditory support to users of ODR (e.g., Hao et al., 2024; Orynbay et al., 2024; Hemavathy et al., 2023), or (Pooja et al., 2024). Typically, TTS is used in daily digital communication to convert written text into spoken words, making it easier and faster to consume information (by simultaneously hearing the words). This technology has many practical applications (see Table 1, lower part), including helping people who are unable to read (or have difficulty in reading) to access written content.

Fortunately, recent advancements in modern deep learning (DL) techniques have improved our ability to perform these tasks. Recent developments in ODR technology use improved DL models such as R-CNN, SSD, and YOLO that are more accurate and faster (see Supplementary Table 1). These models are trained on large datasets of annotated images, such as the COCO (Common Objects in Context; COCO, 2023) and the ImageNet (ImageNet, 2023) collections and are capable of detecting objects with high accuracy in real-time (RT). Characteristically, the COCO dataset contains photos of almost hundred object types, whereas the full ImageNet contains 20 K+ categories (see COCO, 2023; ImageNet, 2023; Lin et al., 2015; Russakovsky et al., 2015; Deng et al., 2009). Moreover, several ODR and TTS algorithms are available today as libraries of commonly used programming languages (Supplementary Table 2 lists some such popular ODR and TTS options; in Python; Python.org, 2023). Several of those libraries offer a set of pre-trained models for ODR making it easier nowadays for developers to implement own algorithms and custom applications [e.g., TensorFlow (2023) or OpenCV (2024)]. Similarly, TTS libraries make it easier to generate speech from text data by offering a variety of features, including the ability to customize voice parameters, adjust speaking rate, and control pitch and volume, as well as conversion in multiple languages [e.g., like pyttsx3 (Bhat, 2021); gTTS (Durette, 2022), and espeak (Asrp, 2024)], making them versatile tools for developers who need to create speech-enabled applications.

Main ways to combine ODR and TTS into integrated speech synthesis systems of spoken descriptions include the:

- Stepwise approach: first using ODR models that output bounding boxes, and then using TTS modalities to convert object labels into speech, or
- Descriptive approach: using ODR models that output detailed information about objects (such as size, shape, or color) and then use TTS systems to generate more detailed spoken descriptions, or
- Hybrid approach: creating single, end-to-end models that are specifically trained to directly output spoken descriptions of detected objects, eliminating the need for combining separate components; this approach can built on the ‘multi-modal’ capabilities that more recent AI increasingly enables—allowing to input one modality (e.g., video or text) and output another (e.g., image or audio).

In comparison, the first approach is more straightforward and easier to implement, but the spoken descriptions may be limited to object labels only. The second approach generates more detailed descriptions but may require more complex ODR models. The third approach has the potential to be more efficient and accurate, but it requires more complex training, which could be a limitation for some programmers developing real-world applications and may not be as interpretable as the other two approaches.

Considering these advancements, we reflect on how effective could a generic solution be today, that is able to transfer key visual information into audio communication. Importantly, such a general solution should be able to apply also in RW and for any language.

To describe this setting, we decided to use the broad phrase ‘*au*dible *r*e*a*lity’ (or AuRa) to denote a variety of options related to using sound perception as a means of experiencing or understanding reality. While this term is not a widely recognized (or commonly used) term in mainstream language or technology, we want to distinguish it from related topics, such as auditory analysis, virtual acoustics, binaural audio, sonification, and so on. Like virtual reality (VR) and augmented reality (AR) create a spatial sense of presence in a (digital) world, AuRa encompasses the use of sound to represent and interact with the physical world. However, in contrast to VR, AR or other mixed reality technologies (e.g., Real and Araujo, 2021), AuRa does not intend to be immersive or to represent digital environments. Moreover, we are interested in (AuRa) solutions that are portable and/or wearable (e.g., Kuriakose et al., 2023; Liu et al., 2018; Wang et al., 2021; Real and Araujo, 2019), without requiring multiple devices, interfaces or advanced neuroscience components (see Wang et al., 2021; Schinazi et al., 2016). Nonetheless, the AuRA solution we envision should be straightforward and able to be used together with other independent wearables referring to further sensory options (e.g., Kilian et al., 2022; Zhu et al., 2023).

**Figure 1:**
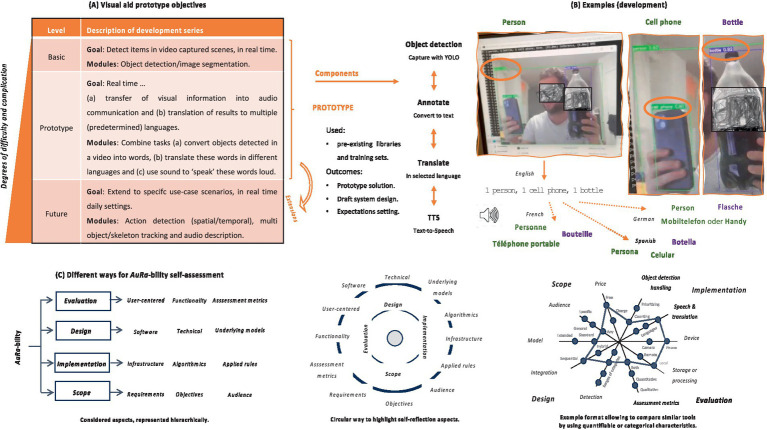
Summary of our approach and results. To build our visual aid system, we focused on being able to annotate image (video frames) in real-time (see, upper left part). **(A)** To do this we use object detection modules that can identify and locate objects within an image (or video), and output bounding boxes around those objects along with a text label that specifies the class of each object. We then automatically extract the names of these categories and pass them though a text-to-speech (TTS) module. Eventually, the generated summary can be ‘spoken’ in voice, in different languages (see, upper middle part). **(B)** Examples of tests during development (see, upper right part; three detected items translated in voice in selected languages)—developer’s face and bottle’s brand blurred. **(C)** Similar implemented technologies can be tested by users in real world settings and their *AuRa*-bility can be summarized in different self-assessment formats (see, lower part).

During this work we also prototyped a proof-of-concept (PoC) and searched for key characteristics to evaluate AuRa performance in RW (see Figure 1). Our PoC was aimed to be deployable also on a smartphone with camera and be able to support users from diverse backgrounds in RT (including both visually impaired individuals and not). Based on this experience, we discuss relevant perspectives (potential, limitations, and challenges) and search for options available today. Importantly, we present a simple way to characterize similar solutions in a self-assessment reflection summary (see Supplementary Table 3). We anticipate that our work will add to the efforts of the community toward the development of more effective and accessible aids, particularly for individuals with visual difficulties.

## 2 Materials and methods

To build our PoC, we combined modern DL components in an integrated solution using ODR and TTS modules in a single pipeline (see Supplementary Data sections 2.1, 2.2, 2.3). Figures 1A,B provide an overview of the whole process.

## 3 Results

We wanted to implement functionalities that can be important in numerous occasions. In specific, we utilized modern DL techniques toward a PoC that could:

- capture main objects present in live video stream frames,
- announce them in voice, by
- using the user’s language of choice.

While this modular approach has been examined previously (e.g., in specific or in limited settings like in Kuriakose et al., 2023; Tapu et al., 2017; Guravaiah et al., 2023; Makhmudov et al., 2020; Alahmadi et al., 2023; Chen, 2022), or (Vijetha and Geetha, 2024), there are not many tools available today that combine these tasks together toward a complete solution that is suitable for the broad audience, for any language. There are several reasons for this dearth, including perhaps commercial prospects and restrictions, amount of effort required, access to resources, as well as maturity of underlying DL models and the rapid changes in the AI landscape.

We expect that as more developments take place, a multitude of programmable options will be examined more consistently spanning both application and user design options (e.g., language selection that the text will be translated into, rate at which the video is sampled or how frequently should the speaker summarize the view, techniques or rules for determining the spoken description, how many and which objects should be prioritized as important, text transformations, output features, or end of process), as well as technical capabilities (e.g., visual capture ability, number of objects and categories that can be detected, view resolution, refresh speed, distance, disk size, memory, etc.). We find that systematic examining of inherently underlying limitations and tradeoffs is important to determine an ‘optimal, default’ setting, especially when it comes to application in non-controlled conditions.

The main difficulty lies in the fact that features that may severely impact functionality and user experience include both quantitative and qualitative aspects that are not easy to measure. Some such examples include input capture (e.g., camera focus or distance of objects may sometimes limit usefulness of live, open world application), accuracy (e.g., errors in ODR, not natural-sounding or easy-to-understand TTS output), performance (e.g., coordination of object capture, detection, and vocal description speed can be a bottleneck leading to out-of-sync visual and audio), possible underlying model bias (e.g., training datasets may not be diverse enough to account for the desired RW scenarios, models may be biased toward recognizing certain types of objects only or toward providing only few speech patterns), hardware (e.g., RT requirement may limit availability and use in certain devices or environments), but also context and understandability aspects (e.g., not all objects may be detected in complex scenes or in scenes with occlusions, TTS models may not always be able to generate speech for a given context, and so on).

Fortunately, modern DL developments provide constant improving to each of those individual aspects and several commercial AI efforts exist, dedicated to relevant tasks (e.g., for video transcription or TTS generation, Video Transcription, 2024; VEED.IO, 2024; LOVO AI, 2024; AI Voice Generator, 2024a; AI Voice Generator, 2024b; Murf AI, 2024; Synthesys.io, 2024; ElevenLabs, 2024; Home, 2024; AI Studios, 2024; Fliki, 2024; Resemble AI, 2024; Descript, 2024; Maestra, 2024; Sonix, 2024; Media.io., 2024; Dubbing Tool, 2024, and so on). However, to put such capabilities in perspective together, in the context of an AuRA scenario is not straightforward. For this reason, we compiled a set of such characteristics that can be considered in combination when it comes to reflecting on the *AuRa*-bility of a tool under development (see Supplementary Table 3). Figure 1C provides a high-level summary of some of those aspects, either in taxonomy or circular format. Importantly, we anticipate that our summary will facilitate a more straightforward comparison and on par evaluation of AuRA tools. Moreover, it offers flexibility in its usage, allowing for the highlighting of different aspects each time. For instance, one might choose to summarize selected measures using a matrix or tabular form (with text- or color-coded cell descriptions), whereas for others a radar or spider representation may be more appropriate (see Figure 1C).

We find that to properly assess the use of current and future (e.g., multimodal) AI/ML based models in open world requires the development of appropriate benchmarks [(e.g., Liu et al., 2018)], probably tailored to specific use-scenarios. Another central source in assessing RW performance of any one integrated AuRa implementation can be direct user evaluation, helping gather essential qualitative feedback. For example, a relevant questionnaire could help determine which customizable parameters are better according to users’ preferences, whether users need training, what functionalities meet best the needs and of each target group, what is the scope of expectations (e.g., regarding input, language, understandability, or speed options) and, importantly, allow to vote what objects and actions/activities are important to be captured (see Supplementary Data section 2.4). We find that such surveys are necessary to determine the development of these systems further, providing timely feedback to improve and extend in terms of RW application.

## 4 Discussion

Inspired by the capabilities enabled by modern AI developments, we sought to explore the potential of transferring key visual information into audio communication toward the development of an audible visual aid. We approached this question from a ‘higher-level’ perspective aiming for fast, portable solutions that can apply in RW, in different languages and in RT. Without neglecting the extensive work done in the broader field, we avoid engaging in extensive historical review or performing in-depth comparison and analysis of previous approaches and current AI developments. Instead, we shortly review available options today and search for pragmatic approaches to combine recent ML/DL models with the objective of building ‘light’ standalone companion-applications that can support situations when visual limitations may hinder the ability to interpret the world.

One key setting where this would be important is for individuals with visual impairments who often face challenges in navigating their environment, in identifying objects, and in interacting with their surroundings. ODR technology has demonstrated potential in tackling some of these difficulties by automatically recognizing and localizing objects within digital images and videos (see Supplementary Table 1). However, interacting with such information alone may be limiting for some, as it may not be easily comprehensible. Speech-based interfaces, on the other hand, provide an additional format that may allow communicating information about objects in the environment (see Table 1), which might not be completely discernible through visual cues alone (e.g., Kuriakose et al., 2023). Combining, thus, these technologies can help improve quality of life both in terms of independence and safety.

Ultimately, automated content capture from live image/video streaming and converting this into spoken descriptions can have numerous important uses. Few such implications include, among others, the improved ability to:

- Navigate unfamiliar indoor/outdoor environments, by detecting obstacles and providing audio feedback on surroundings (e.g., by notifying the user of objects in near proximity in RT, while they are walking).
- Monitor for safety issues, by detecting and alerting the user about potential hazards (such as approaching vehicles, pedestrians, or other objects tracked) the system can help prevent avoidable accidents.
- Enhance independent life capabilities, by interacting with the surroundings more confidently and by performing everyday tasks (such as household activities) more effectively.

Overall, such systems are applicable in several business, industrial and other settings addressing important problems (see also Table 1; Supplementary Table 4), such as:

- Autonomous vehicles (e.g., automated detection of obstacles or of traffic conditions on the road to alert passengers or other vehicles in vicinity).
- Law, security, and surveillance (e.g., detection of people or objects in a specific area to provide alerts or instructions to security personnel).
- Smart homes (e.g., recognizing specific objects or people to provide personalized message greetings, alerts, or instructions to residents).
- Art, entertainment, and education (e.g., RT transcription and translation of visual content to make it more accessible and easier to understand for a wider range of people).

However, in this work we are interested in the support of individuals in cases where visual input may not be otherwise available. Such cases do not only include visual impairment, but also potential emergencies and scenarios for those who prefer speech to text-based interfaces (e.g., due to literacy or language limitations). Currently, there exist several options available for the general public that support similar cases. Supplementary Table 4 lists some such platforms for a variety of tasks (from nutrition to plants): characteristically, Google Lens (2024) has today 10B+ downloads, whereas Narrator’s Voice (2024), Voice Aloud Reader (TTS) (2024) or T2S (2024) have 10 M+ downloads each. Typically, these systems tackle the two tasks (ODR and TTS) mostly separately. More importantly, we find that many of these apply in rather comfortable (controlled) situations, or on restricted settings, and are triggered mainly on demand. Even though this applies also for mobile apps that support visually impaired individuals, we notice that the latter are in comparison reasonably more adjusted for dynamic use in RW, providing RT feedback in non-specific environments (see Table 2).

**Table 2:** Popular mobile apps with many downloads that combine object detection and TTS in real-time to support visually impaired^(+)^.

Name	Downloads^D^	URL	Citation
TapTapSee^B^	500 K+	taptapseeapp.com	TapTapSee (2023)
Lookout^A^	500 K+	goo.gle/lookout	Lookout (2024)
Envision AI^B^	100 K+	letsenvision.com	Envision (2024)
Sullivan+^B^	100 K+	mysullivan.org	Sullivan Plus (2024)
Seeing AI^B^	50 K+	seeingai.com	Seeing AI (2024)

^(+)^More similar apps exist to support visually impaired with less than 100 K downloads such as Supersense (Supersense, 2024) or MyEyes (MyEyes, 2024), whereas some efforts may no longer be available for download and use, or may not be updated regularly [e.g., Vision (Vision, 2024) or Aipoly (V7 Aipoly, 2024)]; also, available options may be different depending on geographical regions. K, denotes thousands; TTS, short for text-to-speech; ^D^information available from Google Play on April, 2024; ^A^available only in Android; ^B^available both for Android and iOS.

All apps examined in Table 2 can recognize objects, people, and texts. Some offer more specific options such as describing color and lighting conditions, identifying currency, or locating objects (e.g., TapTapSee, 2023; Lookout, 2024), or (Seeing AI, 2024). However, we find that RW functionality may in some cases be semi-dependent in that user interaction is required to activate (or to determine respective) tasks after the initial launch of the application. This may be cumbersome in some situations, hindering full autonomy and may require a controlled setting or assistance from (or cooperation with) another person for optimal performance. Moreover, we find that more advanced features (like exploring surroundings, describing scenes or emotions, recognizing specific persons, understanding handwriting, determining approximate distance, or using audio AR to navigate in the world) are often in ‘experimental’ or ‘beta’ mode, requiring more improvement and research (e.g., Liu et al., 2018; TapTapSee, 2023; Lookout, 2024; Seeing AI, 2024).

Despite these limitations, it is expected that the constant technological progress observed today (e.g., updating pre-trained AI models, with more data or new architectures) will help make novel features available soon. Some forward-looking options may include also the repurposing of the multimodal capabilities of modern, integrated large language model (LLM) approaches [e.g., such as OpenAI’s GPT-4 (GPT-4, 2024), Anthropic’s Claude (Claude, 2024), or Google’s DeepMind Gemini (Google DeepMind, 2024) models among others]. While direct human feedback remains the best in aiding the visually impaired, virtual AI enabled modules already complement such services that connect people needing sighted support with volunteers (e.g., Be my eyes, 2024) offers a virtual assistant integrating automated image-to-text technology and OpenAI GPT-4 features (OpenAI’s GPT-4, 2024). Regarding design, we find that most Table 2 apps face similar challenges, irrespective of their AI modalities—examples include:

- World setting and input quality (e.g., each smartphone app is only able to recognize objects that are in focus and within the camera’s scope; lighting conditions are also important for the quality of the identification).
- Visual capture (e.g., use of the phone’s camera to take a picture or a video; some require the activity to take place upon demand, whereas others require slow speed while moving to allow for RT assessment, like Lookout, 2024); video stream might be limited in size—e.g., TapTapSee allows for videos that are up to 10 sec long to be captured each time (TapTapSee, 2023).
- Functionality grouping (e.g., whether the task is concerned with texts, objects, people, or other specific goal).
- Activation method (e.g., depending on the app, the task at hand—whether video capture or image analysis—could be triggered in different ways, like by tapping or via voice command; e.g., TapTapSee, 2023; Sullivan Plus, 2024).
- Cloud based services (i.e., some require online access to work; e.g., TapTapSee, 2023; Seeing AI, 2024).
- Multi-lingual support [i.e., number of languages that can be used may vary depending on task or on phone’s OS—e.g., Seeing AI supports 20 languages (Seeing AI, 2024)], whereas Envision AI can read up to 60 languages (Envision, 2024); other apps use the language setting of phone’s OS, like Lookout (2024).

Finally, even when users change language settings, this may not apply equally well for all languages or to all tasks [e.g., Lookout (2024) has a separate functionality for text reading and for food labels]. As result, some features may not be available in all languages or may perform better in some than in others. Nevertheless, multilanguage support is constantly evolving and we expect that this gap will close over time. One characteristic example of relevant rapid developments in recent years is the growth of LLMs, including their ability to translate between languages—some notable LLM models include Google’s Bert (see BERT, 2024; Devlin et al., 2019; Open Sourcing BERT, 2024; Google-research/bert, 2024), T5 (see T5, 2024; Raffel, 2023; Python. Google Research, 2024; Google-research/t5x, 2024), LaMDA (see Google, 2024; Thoppilan, 2022), PaLM (Google AI, 2024) and the more recent Bard (now Gemini, 2024), OpenAI’s GPT series (v4, GPT-4, 2024), Anthropic’s Claude (Claude, 2024), Microsoft’s Copilot (Microsoft Copilot, 2024), Meta’s LLaMa family (see Meta Llama, 2024; Meta-llama/llama3, 2024) and Mistral’s AI models (Mistral AI, 2024). Uniting efforts away from a suite of AI language translation models toward a single speech model supporting multiple languages is endorsed by several larger corporations and open source contributions, like Meta (e.g., Meta AI, 2024a; Pratap, 2023) and the No Language Left Behind (NLLB) initiative (see Meta AI, 2024b; NLLB Team, 2022; NLLB, 2024; NLLB-MOE, 2024).

Ultimately, RT video object descriptions should provide valuable information in a variety of settings, not only to support individual’s decision making in daily life but also to improve safety, quality of care, efficiency, and social inclusion (see Table 3). The development of such applications can benefit not only visually impaired persons but also other individuals who may have difficulty interpreting visual information, such as individuals with cognitive disabilities, with language or social barriers, or with limited literacy skills. Table 3 mentions few such cases when accessible AuRa experience can be important, with potential applications spanning various fields, including healthcare, education, and entertainment.

**Table 3:** Potential AuRA applications to personal and community well-being.

Area/Topic	Description (examples, scenarios)^H^
Social equality and integration	There are several groups of people who would benefit from real-time audio description, promoting participation and interaction. Key audience is people with visual disabilities who require more accessible and understandable content. Examples: Social media: generated high-quality audio transcripts can assist in creators and content becoming more accessible to a wider audience. Politics and businesses: marketing, advertising, and e-commerce content can become more accessible and appealing to a wider audience. Family and friends: enabling of team-based activities with independent participation by overcoming sight limitations.
Arts, culture, entertainment	There are several groups of people who would benefit from real-time audio description, enhancing both experience and understanding. Examples: Description of landscapes, statues, paintings, architectures. Audible captions for live events, such as public speeches, presentations, and other visual broadcasts; applies to theater, museum, cinema, video gaming as well as outdoor activities.
Education and research	Consider researchers and educators who want to create searchable content for easier reference and analysis, or learners interested in understanding content for educational purposes. Examples: Audible captions for live events, such as public speeches, presentations, and other visual broadcasts, like in classroom or scientific conferences. Children learning the names of objects (in own or other language). Opportunities for multilingual learning play and simulation-based training.
Health and safety	Remote counseling in isolated places or in limited visibility settings (e.g., due to distance, geographical or extreme weather conditions). Examples: Clinical operation: healthcare professionals to identify and track medical equipment and supplies in remote site in real-time. Telesurgery: audio confirmation of tools available on-site during operation progress. Child safety: description of child’s environment (identification of harmful objects) to avoid accidents, improving their independence, confidence, and mobility.
Emergency response	Remote support (e.g., via drones) to help efficient and effective search and rescue operations (e.g., fires, floods, earthquakes), ultimately saving lives and reducing the risk to rescue workers. Examples: Provision of valuable information, improving response times, safety, and chances of survival. Quick identification and localization of critical equipment, supplies, and victims or individuals who need assistance, in real-time, and in low-light or obscured environments. Provision verbal instructions to individuals who may be disoriented or unable to communicate effectively (e.g., help a person in distress understand the situation by providing information while waiting for rescue). Audio description of drone imagery transmitted to rescuers regarding the source or identification of hazardous materials in a critical area—gathering of information and assessment of the situation while in another area from safe distance, reducing exposure to danger.

^H^Highlighted scenarios are intended to refer to circumstances when audio (listening) is more admissible than visual (or other sensory) mediums. Examples of such circumstances may include multitasking, lack of personnel, or remote monitoring (e.g., consider a first responder with broken microphone transmitting live video to another field coordinator whose display-monitor has been broken).

While the simplest AuRa form can thus be potentially of interest to a wide range of circumstances, in any setting in which a user’s main input sensor is hearing (from remote device controlling to the safety monitoring of children or pets), it can be easily combined or enhanced with additional or with more advanced technologies (e.g., VR/AR extensions may also apply).

Nevertheless, despite significant progress, there are still challenges to address. One challenge is the need for robust ODR that can accurately perform in a wide range of environments and lighting conditions. In that aspect, we believe that modern generative AI techniques have the potential to be effective in addressing cases of fuzzy image capture in open world (e.g., by enhancing resolution or by generating simulated frames in incomplete video). Another challenge is the need for natural and expressive TTS systems that can convey object information in a clear and understandable manner. Beyond the stepwise approach and basic architecture of our PoC, there are today several opportunities to benefit from current multimodal breakthroughs [such as GPT-4, 2024, Claude, 2024, or Gemini (Google DeepMind, 2024)] that can handle both text and vision inputs, or the other way round (e.g., Imagen, 2024, Parti, 2024, DALL·E3, 2024, Sora, 2024), Make-A-Video (Meta AI, 2024c), Gen-2 (Runway, 2024), or Lumiere (2024). However, this progress must also be considered with caution, especially when it comes to specific tasks [e.g., see intricacies in LLM performance regarding scientific context, like Galactica Demo (2024) and the biochemical domain (Zhang, 2024)] or modalities, and it is unclear how much of AuRability such united AI efforts could address today already. We find that more user-centered design and evaluation studies are needed to ensure that the needs and preferences of users (whether visually impaired or not) are adequately met. Direct comparisons of such tools are also not straightforward and may have to consider several dimensions (e.g., Kuriakose et al., 2023; see also Figure 1C; Supplementary Table 3; Supplementary Section 2.4).

With our prototype experience discussion, we do not attempt to make a new proposal, but rather to reflect on the applicability of this given architecture and stepwise approach. We want to draw the attention toward the creation of better performing solutions and to establish an easy-to-use, adaptable, and transferable setting for extended use by the broader community. We believe that personalization and adaptations tailored to the specific circumstances of potential users will be warranted in future. For example, many of the freely available software (open) libraries and models today (e.g., see Supplementary Table 2), are pre-trained with ‘relatively limited’ image datasets—e.g., the ‘COCO’ dataset (COCO, 2023) is composed of only less than hundred different object classes (cars, persons, sport balls, bicycles, dogs, cats, horses, etc.)—that may be too generic for specific tasks. On the other hand, determining extended image datasets (e.g., ImageNet, 2023) should consider also the level of detail or abstractness that respective category labels will be described with. For example, using specific only dictionaries or ontologies, might interfere with effective multilingual support since some terminology or words may not be obvious to (unambiguously) translate in any language. Dictionary independent translation and TTS models—from any language to any language [e.g., NLLB (Meta AI, 2024b)]—are therefore important to be considered, especially when it comes to capturing titles of actions or of activities. From this perspective, action recognition poses challenges not only regarding technological implications (e.g., input, DL architecture and datasets), but also regarding the types (and number of predefined) action classes that should be (adequately) recognizable. Automated AI enabled ‘audio description’ projects require also further attention and standardization, especially when it comes to low vision users, to content that is not expressed via sound (e.g., a dialog) and to the diversity of possible context settings [e.g., Kuriakose et al., 2023; Brack, 2024; AD Lab Pro, 2024; Web Accessibility Initiative (WAI), 2024; Wang et al., 2021; Van Daele et al., 2024; Jain, 2023; Ning, 2024]. On some occasions, haptic, physical, or hardware support may contribute to improved detection and description performance (e.g., via marked labels or fixed QR codes placed at determined locations helping the AuRa system). Direct community support may also largely help extend AuRabilty scope (e.g., by engaging in image labeling activities, by prioritizing actions or objects deemed important to be handled first in different scenarios, and so on).

In future, we expect implementations with optimized sub-components and extended functionalities that revolve around (a) the decision-making support (e.g., for avoiding obstacles) and (b) the description of activities captured in longer, continuous ‘single-take’ video stream frames. These may also come as sophisticated versions of ‘hybrid’ approaches (e.g., integrated multi-modal systems)—indeed, present-day state-of-the-art provides a lot of opportunity to timely explore the extent that such capabilities can catalyze AuRa applications, contributing to several improvements that range from more efficient management of lengthy content (e.g., handling longer durations) to more advanced question answering and complex understanding skills (e.g., suitable for expert or domain-specific application). Ultimately, an advanced live AuRa description system will be an extended solution that can generate (sub-) title like audio text descriptions (or question responses) to provide an augmented experience.

To set priorities of future developments more appropriately, we invite the community and the public to engage in organized feedback projects (e.g., via questionnaires) providing regularly structured information guiding the specific goals and user requirements (e.g., about input parameters, languages, speed, understandability, scope, functionalities, types of objects, device components, etc.) that newer, targeted solutions should address. We also expect that such efforts can be more efficiently supported by the active involvement of the community in the preparation of datasets (e.g., training examples) from the collective contribution of crowd collaboration projects. Several platforms exist today that can enable exchange of image labeling information and annotation initiatives (e.g., Make Sense, 2024; V7, 2024; Dutta and Zisserman, 2019; CVAT, 2024; Open Source Data Labeling, 2024; Dataloop, 2024; Data Labeling, 2024; SuperAnnotate, 2024; Encord, 2024; LabelMe, 2024; Roboflow, 2024).

Altogether, we find that a straightforward architecture comprised of four main steps (i.e., video capture, object identification, description in text, translation in different languages) is a generic approach capable today already to help with the goal of building a working AuRa framework (see Figures 1A,B). With our work, emphasize, in addition, the importance of soliciting feedback directly from potential target groups to better guide tailored preferences and to inform future developments (see also Supplementary Table 3; Supplementary Section 2.4). To our opinion, the field can be dealt with more systematically, particularly given the technological capabilities demonstrated recently. In strong support of this direction is also the example of Project Astra (2024), a very freshly released initiative by Google’s DeepMind toward a universal digital AI assistant. We believe that the community, even when with limited resources, should not miss the chance to more actively aid, together with larger organizations, in the structured evaluation and benchmarking of such advanced AI agents that are capable of more complex reasoning and multimodal interactivity. For these reasons, we anticipate that our discussion will be seminal, influencing some of the coming efforts of the community toward the development of more effective and accessible digital solutions for visually impaired persons, but also inspiring tools for important applications in medical, health or other emergency settings (see also Table 3). Importantly, our discussion underscores the role and impact of such digital interventions in protecting and improving broader public health and policies in terms of both personal and community well-being.

## 5 Conclusion

Combining object detection and speech conversion technologies has the potential to significantly enhance accessibility of information for visually impaired individuals. Beyond integrating separate distinct modules, we envisage more dynamic, open world applications, performing in RT, for any place and language. Many of today’s portable mobile solutions are potentially able to contribute into breaking both visual impairment inequalities and restrictive language barriers. While there are still challenges to be addressed, the progress made in this area has been significant, and there is a strong foundation laid for continued development and optimization of these technologies. The community should also take advantage of the opportunity to explore the possibilities enabled by repurposing modern AI advancements (multimodal capabilities, improved interactivity, and more complicated reasoning) to tackle everyday situations. In addition, we expect that in near future more studies will take place to examine underlying trade-offs and that coming tools will enable functionalities that are tailored to more specific scenarios and audiences (e.g., imagine an AuRa agent answering to a visually impaired person reliably whether a street is safe to cross at a certain moment). We, therefore, invite the community to gather this information in an organized manner and to create appropriate performance benchmarks, which can inform decisions regarding model selection and system optimization strategies. We anticipate that broader, collaboratively sourced feedback can serve as an effective guide to the characteristics of future data focus and training efforts. Finally, we aspire that our comments and discussion will help raise more awareness on the challenges of visual impairment as well as will be influential to multiple such efforts.

## Data Availability

The original contributions presented in the study are included in the article/Supplementary material, further inquiries can be directed to the corresponding author.
