# Supplementary material for: A brief reference to AI-driven audible reality (AuRa) in open world: potential, applications, and evaluation
Source: Front Artif Intell. 2024 Oct 25;7:1424371. doi: 10.3389/frai.2024.1424371 (PMC11543578; doi:10.3389/frai.2024.1424371)
Supplement: Supplementary file 1 [file Data_Sheet_1.pdf]

Supplementary Material

A brief reference to AI-driven audible reality (AuRa) in open world: potential, applications, and evaluation.

Ömer Ates, Garima Pandey, Athanasios Gousiopoulos, Theodoros G. Soldatos *

* Correspondence

1 Supplementary Tables

This section lists four Supplementary Tables supporting the main text.

Supplementary Table 1. Deep learning (DL) algorithms offer multiple solutions in key computer vision and text-to-speech (TTS) tasks.

Task	Examples of key modern algorithms
Object recognition	NN [NN]-based solutions where the input is pictures, and the output is a classification or localization of the object(s) in the image. Examples of algorithms include RCNN [R], Fast RCNN, Faster RCNN, Mask RCNN, SSD [SD] and the YOLO [Y] series.
Image classification	NN [NN]-based solutions where the input is pictures, and the output is probability of belonging to a category. Examples include the VGG [VGG], Inception and ResNet [RN] series.
Semantic segmentation	DL models that classify and group each pixel by creating a segmentation mask. Examples of algorithms include the FCN [F], U-Net [U], SegNet [SN] and the DeepLab family of models.
Video understanding	Key video understanding tasks include video classification and behavior detection. Examples of algorithms include C3D [C], TSN [T], DOVF [D] and TS_LSTM [TL].
Text understanding (and OCR [O])	In text detection the region that mentions text is indicated by a surrounding box, whereas in text recognition the detected region is further processed in order to recognize what the text is. Examples of algorithms include MSER [M], EAST [E], CRAFT [CR], and CRNN [CN].
Object tracking	Detected object is indicated by a surrounding box and assigned unique id for tracking as it 'moves' through frames while storing the relevant information. Examples of algorithms include KCF [K], DEFT [DT], CenterTrack, or ODESA [OD].
TTS	The goal of TTS is to convert written text into spoken words by generating a synthesis of high-quality, natural-sounding speech from the input. Examples of algorithms include Tacotron, WaveGlow and the WaveNet, Deep Voice, and FastSpeech series.

Abbreviations: [NN] neural network; [VGG] visual geometry group; [RN] residual network; [F]: fully convolutional network; [U] U-shaped architecture; [SN] segmentation network; [C] Convolutional 3D; [T] temporal segment network; [D] deep one-vs-five; [TL]: temporal segment based long short-term memory; [O] optical character recognition; [M] maximally stable extremal regions; [E] efficient and accurate scene text; [CR] character region awareness for text; [CN] convolutional recurrent neural network; [K] kernelized correlation filter; [DT] detection embeddings for tracking; [OD] object descriptor that is smooth appearance-wise; [R] region-based convolutional neural network; [SD] single shot multi-box detector; [Y] you only look once.

Supplementary Table 2. Popular libraries and packages commonly used for object recognition and TTS tasks, available in Python.

Task [T]	Name: description
Object recognition	• **TensorFlow**: platform that provides tools for building and training object detection models, as well as a framework that allows developers to create custom models using pre-trained networks. Latest release: 2.16.1; URL: tensorflow.org<br>• **OpenCV**: the Open-Source Computer Vision (OpenCV) library offers a wide range of functions and pre-trained models for object detection and other computer vision tasks (such as face recognition, image segmentation, etc.). Latest release: 4.9.0; URL: opencv.org<br>• **Scikit-Image**: collection of algorithms and tools for image analysis and manipulation. Latest version: 0.23.1; URL: https://scikit-image.org | See also [(1)].<br>• **PyTorch**: framework providing an ecosystem of deep learning libraries, such as computer vision tools. URL: https://pytorch.org<br>• **GluonCV**: toolkit of computer vision implementations, over *Apache MXNet*, for rapid prototyping and experimentation. URL: https://pypi.org/project/gluoncv | See also [(2)].<br>• **ImageAI**: open-source library to help simply building computer vision applications. Latest version: 3.0.3; URL: https://pypi.org/project/imageai | See also [(3), (4)].<br>• **Detectron2**: platform for object detection, segmentation and other visual recognition tasks, over *PyTorch*, by Meta [FAIR]. URL: pypi.org/project/detectron2-cdo | See [(5), (6)].<br>• **OpenMMLab**: community based open-source toolbox ecosystem of projects covering a wide range of computer vision algorithms. URL: https://openmmlab.com
TTS	• **gTTS**: library for interfacing with Google's Text-to-Speech (gTTS) API service. Latest version: 2.5.1; URL: https://pypi.org/project/gTTS<br>• **pyttsx3**: library for offline TTS conversion; cross-platform TTS engine supporting various operating systems, often used for applications where an internet connection is not available or desirable. Latest version: 2.90; URL: https://pypi.org/project/pyttsx3<br>• **talkey**: library providing multi-language support and a simple interface to different TTS engines, including *eSpeak*. Latest version: 0.1.1; URL: https://pypi.org/project/talkey<br>• **tts-watson**: library allowing developers to interface with IBM Watson's cloud-based TTS API service. Latest version: 1.0.0; URL: pypi.org/project/tts-watson | See also [(7), (8)].<br>• **aws-polly**: TTS service provided by Amazon Web Services (AWS), supports multiple languages, some with lifelike speech using deep learning models. Latest version: 0.1; URL: https://pypi.org/project/aws-polly | See also [(9), (10)].<br>• **espeak**: a compact, cross-platform, free and open-source TTS engine that supports multiple languages, often used in applications where a lightweight and portable TTS solution is required. Latest version: 0.6.3; URL: https://pypi.org/project/python-espeak | See [(11)].

Notes: [T] The table contains a select list of characteristic examples and is not exhaustive. For example, some TTS engine or libraries (like Festival (12), (13) or Pico (14), (15)) are not included, nor does it include image processing and manipulation libraries (e.g., see SciPy (16), or Pillow (17), (18)) or MLOps platforms designed to facilitate machine learning (ML) experiments or manage ML projects and pipelines, like MLflow (an open-source platform with a focus on managing the ML lifecycle, (19)) or Neptune.ai (a cloud-based platform with a focus on experiment tracking, visualization, and collaboration, (20)); [FAIR] Facebook AI Research.

Supplementary Table 3. Summary of *AuRa*-bility aspects organized in four (4) main dimensions. The listed features are not exhaustive and serve as examples that we aim to extend with the help and feedback of the community.

Dimension	Description of relevant aspects
Evaluation	**User oriented:**<br>• **Design** [GUI] **preferences**: refers to the provision of ***options*** for the user to select from, as well to the number of such ***configurable*** options (e.g., available speech voices).<br>• **Usability**: refers to aspects such as ease of understanding and use, user friendliness and accessibility (e.g., for visually impaired users), solution's interactivity and responsiveness, whether previous experience or training is required, and so on.<br>**Functionality**: refers to the underlying modalities – object, detection, recognition, and translation – regarding speed, accuracy, efficiency, security (safety), etc., as well as to real-world performance evaluation (e.g., reliability, user feedback, non-controlled settings).<br>**Assessment metrics**: refers to aspects such as, automated gathering of quantitative measurements or of qualitative feedback (e.g., via user questionnaire), and comparison against other tools.
Technical design (incl. software)	Refers to *technical* **parameters** (e.g., related to ***input***, ***output***, ***system***, ***storage***, etc.) as well as to *underlying model* [DL] **settings**.<br>Examples, of such **characteristics** include choice of language, image or video input, duration of input processed each time, duration of storage of respective capture, dynamic or static capture, remote or local processing, choice of speech voice, app- or web-based interface, degree of text processing (capture, transform, translate), types of output (e.g., speech only, voice and text, other extended form).<br>Other aspects may include degree of object recognition and TTS **integration** (namely, stepwise, descriptive, or hybrid options [TTS]), whether pre-trained models [DL] suffice, whether a 'standard' set of recognized **object categories** (or more) are required, accordingly whether model adjustments, fine-tuning or extensions are necessary, whether 'personalized' training options for **individual** settings are called for (e.g., for specific use scenarios or application circumstances ,such as region, location or weather conditions, decision-making, and so on) and whether respective labels (training examples) are available (e.g., provided by the user).<br>Note that several of these features can be determined by users as ***configurable*** options.
Implementation considerations	Refers to both ***technical*** and ***algorithmic*** aspects.<br>• Technical **infrastructure**: examples include deployment and device settings (e.g., camera or phone), whether use of a local (device) approach and/or of a cloud-based solution, whether remote or local storage, whether captured images/video should be shared further (e.g., externally) and for how long, duration of captured input, whether capture should periodically restart automatically, and so on.<br>• Applied algorithmic **rules**: examples include ***object detection*** handling (such as number of items detected, whether number of occurrences are counted, whether 'top' objects are determined and how, number of categories to be recognized, sampling frequency, number of frames sampled, whether all or a limited 'top' list is forwarded for speech, form of description, and so on), ***speech processing*** and ***translation*** (like selected voice, talking speed, how frequently should the speaker summarize the view, acceptable accents and delay between visual capture and speech), as well as ***output*** criteria (for instance, description detail – e.g.., only listing of items or also number of, color and shape, relative location, etc. – or whether detected items and/or their vocal representation are prioritized – e.g., based on best recognition accuracy, proximity, or amount – towards a decision-making support scheme).

Scope	Refers to **expectations**, for instance, regarding:<br>• **Requirements**, such as price (whether the service provided is free of charge or available for a fee, integration capability (i.e., how well and easy can it combine or extend with other available tools or technologies) or whether WiFi [NET] is necessary (e.g., for remote or cloud transaction).<br>• **Objectives**, like whether complex environment handling is expected, whether indoor and/or outdoor use, or whether decision-making support is included (e.g., warning).<br>• **Audiences:** namely, main target groups and user focus (e.g., individuals, families, educators and learners, emergency (first) responders, visually impairment or not, and so on).<br>• **Advanced** detection or speech **options**, like captioning or elaborate description details (e.g., including additional features like action, closeness, distance, color, shape, etc.).

Abbreviations: [GUI] Graphical User Interface; [DL] Deep Learning; [TTS] Text-To-Speech combining (options as described in the main text); [NET] wireless (or other) internet connection.

Supplementary Table 4. Ten free mobile apps that provide object detection and text-to-speech (TTS) technology to non-experts in the public, listed in descending order of downloads.

Task [+]	Name	Description
Object detection	Google Lens	Utilizes image recognition technology regrading objects, labels, text, QR codes, places (locations, sights) and retrieves and displays a multitude of relevant information. Downloads: 10B+ | URL: lens.google (21)
	CamFind	Picture based visual search engine understanding displayed content to support user-friendly functionalities. Downloads: 5M+ | URL: camfindapp.com (22)
	TapTapSee	Mobile camera assistive technology for blind and visually impaired, providing in voice object identification results from images or short videos. Downloads: 500K+ | URL: taptapseeapp.com (23)
	LeafSnap	Identifies plant species from input image and provides step by step care guides, information, and recommendations. Downloads: 1M+ | URL: leafsnap.app (24)
	Calorie Mama	Food recognition and nutrition profiling from smart camera images. Downloads: 100K+ | URL: caloriemama.ai (25)
TTS	Narrator's Voice	TTS app to customize narration with a range of languages and sounding effects and voices; can work offline. Downloads: 10M+ | See (26)
	Voice Aloud Reader	TTS to read aloud web pages, news articles, lengthy emails, and multiple different types of text; provides multiple language support and way to input content. Downloads: 10M+ | See (27)
	NaturalReader	Cross-platform TTS for personal, commercial, and educational use; handling multiple text formats. Downloads: 1M+ | URL: naturalreaders.com (28)
	Speechify	Configurable TTS supporting multiple languages and voices as well as input formats, such as images. Downloads: 1M+ | URL: speechify.com (29)
	T2S	TTS for main text file formats; also allows converting to audio file and specifying which part to be spoken, making it simple to listen to a few sentences rather than an

		entire article. Downloads: 10M+ | URL: app-t2s.web.app (30)

Notes: [+] The table is not exhaustive – for example, there exist several apps specific for plant recognition and care guide (e.g., iNaturalist (31), Flora Incognita (32), LeafSnap (24), Plantum/NatureID (33), PlantNet (34), PlantSnap (35), PictureThis (36), FlowerChecker/Plant.id (37), Blossom (38), Garden Answers (39), What's that flower? (40), Garden Compass (41), Plantix (42), Planta (43), not to mention Google Lens itself (21)) and many more for nutrition (e.g., Foodvisor (44), Calorie Mama Food AI (25), SnapCalorie (45), LogMeal Food AI (46), Yummy (47), or Snap It (48) and so on (49)) – and available options may be different depending on geographical location; [D] Information about the number of downloads from Google Play, on April 2024. B: denotes billions; M: denotes millions; K: denotes thousands.

2 Supplementary Data

The following sections support main text content. Presented PoC results are based on concepts discussed during ÖA's and GP's Master's studies, supervised by TS. Selected code from ÖA's Master Thesis can be found at the GitHub repository, see: https://github.com/atesch93/yolov7_last and https://github.com/atesch93/yolov8.

2.1 Software

To build the generic proof of concept (PoC), Python was used to combine different libraries. The latest YOLO (v7 (50), at the time of development) was used for object detection, and the pyttsx3 and gTTS libraries were considered or TTS. PoC development spanned late year 2022 and early 2023 – as such, only few tests considered YOLOv8 (51).

YOLO was chosen due to its speed, simplicity, and key characteristics like high accuracy (uses a global context to predict objects, which considers the entire image, rather than just local regions), efficient multi-object detection (detection in a single pass, predicting the class and bounding box for all objects in an image, simultaneously), and real-time performance (can process images in real-time, which makes it suitable for critical applications where fast object detection is important) (see (52), (50)). Moreover, its approach can be more memory-efficient, as compared to other algorithms that may require more complex pipeline/settings.

Similarly, pyttsx3 was chosen because it can work offline, a property making it suitable for a portable standalone software solution. Google's gtts was interfaced with the googletrans library for translation.

2.2 Architecture

The above elements were combined into a system that takes as input images and videos taken in real-time from the (front) camera of a device (whether laptop or smartphone). This input goes through the YOLOv7 algorithm for object detection. The labels produced at the object detection stage are saved locally in a .txt file and then used as input to the (translation and) TTS module (see main text's Figure 1A and 1B). Both examined TTS modules allow controlling parameters to customize the speech output (e.g., various aspects of speech, including language, speech pace, and pronunciation). Development took place on (personal) device with Intel(R) Core (TM) i7-10750H CPU @ 2.60GHz 2.59 GHz processor; 64-bit operating system; 16,0 GB RAM and

8MP camera; Graphics: Intel(R) UHD and Graphics GeForce RTX 2060; Display resolution 1920 x 1080 at 74 Hz and color depth 32 Bits/Pixels.

2.3 Reference synopsis

To profile characteristics pertaining the development of an effective AuRa solution, we discuss some of our experience and reflect on evaluation aspects. The goal of our effort was to determine parameters that may influence how well a generic solution can deal with two main challenges, namely (a) accuracy in detecting and recognizing objects, particularly in complex environments (e.g., with multiple objects), and (b) usability (i.e., whether the system is easy for users to understand and use; including visually impaired individuals).

For this we considered both quantitative and qualitative options, especially through the prism of real-world performance, in non-controlled settings. Finally, we also attempt to review many of those dimensions in a manually compiled organized structure (see main text's Figure 1C and Supplementary Table 3).

Our PoC experience supports some sensible observations, such as that local processing can provide more easily a robust AuRa interaction, as compared to a cloud/web-based counterpart, that longer video durations may come with increased processing delays, as well as that Yolo-v8 is significantly faster than Yolo-v7, capturing items also with higher certainty. We find that 20 seconds can be a fair length to serve as an initial default threshold (or limit) to process in one interval each time, as video-to-generated-audio synchronization delays may significantly increase for longer live recordings, depending on device and/or application requirements. Moreover, despite YOLO's simple architecture making it easy to implement and to adapt to real world tasks and with higher rates of accuracy, it still comes pretrained with limited data, struggling with small objects and the ability to perform fine-grained classification.

With our PoC experience, we challenged different strategies to make simultaneously possible (a) the support of any (available) language (cross-platform translation libraries), (b) in either online or offline setting (i.e., standalone running, after initial local installation), with (c) continuous live visual capture (where video stream is restarted in short pre-specified time intervals, emptying previous memory cache). In this way we manage to profile an AuRa service suitable for everyone alike (i.e., visually impaired, and not), that can run in rather dynamic fashion (i.e., can apply in an unrestricted setting, is not dependent on surrounding technologies, and is not tailored to specific rules, for some places or situations only). We find particularly important the breaking of the language barrier towards tools suitable also for speakers of non-common languages as well as towards tools enabling learning. However, our PoC is far from a productive state and performs only one task – designed to list with voice any detected items that happen to be in focus within the camera's scope at a given time.

2.4 User feedback questionnaire

During our work we wondered what set of questions might best help extract specific information regarding a broad range of aspects pertaining any given AuRa implementation. However, there is no one distinct way to address this challenging goal and there are many questionnaire and survey design details to consider. While methods used to collect feedback from users may include surveys, interviews, or focus groups, the objectives of such survey activities and the required

output might not be as obvious each time. For this reason, we shortlist a few aspects that we find are important to be considered in such surveys (and sample questions for each):

- **Evaluating use of the implementation**. This group of questions would be concerned with aspects such as:
  - ***Usability assessment***; e.g., Is the AuRa tool straightforward and easy to use?
  - ***Understandability evaluation***; e.g., Does the TTS module provide information in clear language and concise speech?
  - ***Acceptance and applicability***; e.g., Does the tool achieve its goal? Or, is it helpful to individuals visually impaired? Could it be used equally by non-visually impaired individuals, or by both?
  - ***Confidence in usage***; e.g., Do you feel (more) confident when using the tool?
- **Language and object recognition**. This group of questions could be more quantifiable or specific. Altogether they would focus on aspects like:
  - ***Object recognition performance and sufficiency***; e.g., Are the detected objects correct? Are they enough?
  - ***Language importance and correctness;*** e.g., Is the (translated) description correct, or adequate? How important is this for you? Or, is (a specific) language more important to you?
- **Awareness and training**. These questions would address aspects like:
  - ***Training requirements;*** e.g., Do users require (special) training?
  - ***Recommendation;*** e.g., Would you recommend this tool? Would you recommend other tools instead?
  - ***Awareness;*** e.g., Do you know of other (similar) AuRA tools? Do you know any visually impaired people who would benefit from using an AuRa-like tool?
- **Experience and preferences.** These questions would address aspects such as expectations regarding scope, what objects, actions and/or activities are important for the user group – these questions could be more specific each time:
  - ***Background***; e.g., Have you used AuRa tools before? Will you share the AuRA project information with visually impaired people you know? Have you used other similar tools or apps before?
  - ***Exposure***; e.g., Are you currently (or regularly) using other digital visual aids? Have you used augmented or virtual enhancements previously?
  - ***Application***; e.g., In your opinion, in what type of situation(s) would AuRA be most useful? What kind of object(s) would you want AuRA to detect? Do you prefer precision over speed? Would you want more specific scenarios to be address? What would these be?
  - ***Desired interface and implemented feature customizability***; e.g., Do you find AuRA's multi-language option useful? Would you like more items to be recognizable? Which objects do you highlight as priority? What features should be customizable (e.g., language, speed, object prioritization, image sampling, screen capture rate, etc.)? What additional features would you wish implemented?
  - ***Cost (price)***; e.g., How much would you be willing to pay for using this AuRa tool?
- **Advanced considerations**. Aspects pertaining this set of questions would revolve around ***technical preferences*** and ***ethical considerations*** (such as whether data protection is important for the user, whether it is important to understand how the application works, where the data are stored, or whether a preference regarding a local or cloud-based solution exists?).
- **Demographics**. Refers to questions profiling the characteristics of the intended user group (such as age, gender, whether visually impaired, type of profession, education, context of use, and so on).

Overall, we want that the collection of answers are determined by experiences of direct AuRa users (whether visually impaired, educators, or first responders), 'coming from the field' as much as possible. Note that our survey discussion is meant to provide a main initial guideline only and can be adapted, adjusted, or extended to suit requirements best fitting each time. It is also not designed to directly compare different tools, but it could be used for such purposes too (e.g., by contrasting or rating answers about different tools, based on the same questionnaire).

3 Supplementary Figures

None.

4 Supplementary Bibliography

1. Walt S van der, Schönberger JL, Nunez-Iglesias J, Boulogne F, Warner JD, Yager N, et al. scikit-image: image processing in Python. PeerJ. 2014 Jun 19;2:e453.
2. GluonCV Toolkit [Internet]. [cited 2024 Apr 12]. Available from: https://cv.gluon.ai/
3. OLAFENWA M. OlafenwaMoses/ImageAI [Internet]. 2024 [cited 2024 Apr 12]. Available from: https://github.com/OlafenwaMoses/ImageAI
4. Official English Documentation for ImageAI! — ImageAI 3.0.2 documentation [Internet]. [cited 2024 Apr 12]. Available from: https://imageai.readthedocs.io/en/latest/
5. Welcome to detectron2's documentation! — detectron2 0.6 documentation [Internet]. [cited 2024 Apr 12]. Available from: https://detectron2.readthedocs.io/en/latest/
6. facebookresearch/detectron2 [Internet]. Meta Research; 2024 [cited 2024 Apr 12]. Available from: https://github.com/facebookresearch/detectron2
7. IBM Watson Text to Speech [Internet]. [cited 2024 Apr 11]. Available from: https://www.ibm.com/products/text-to-speech
8. IBM/watson-tts-python [Internet]. International Business Machines; 2023 [cited 2024 Apr 11]. Available from: https://github.com/IBM/watson-tts-python
9. Amazon Web Services, Inc. [Internet]. [cited 2024 Apr 11]. Text To Speech AI Tool - Text to Voice Software - Amazon Polly - AWS. Available from: https://aws.amazon.com/polly/
10. cloudyr/aws.polly [Internet]. the cloudyr project; 2024 [cited 2024 Apr 11]. Available from: https://github.com/cloudyr/aws.polly
11. espeak-ng/espeak-ng [Internet]. espeak-ng; 2024 [cited 2024 Apr 11]. Available from: https://github.com/espeak-ng/espeak-ng
12. Robertson P. pyfestival: Python Festival module [Internet]. [cited 2024 Apr 12]. Available from: https://github.com/techiaith/pyfestival
13. Festival [Internet]. [cited 2024 Apr 12]. Available from: https://www.cstr.ed.ac.uk/projects/festival/
14. Bartsch G. py-picotts: Python interface for SVOX Pico TTS pico2wave [Internet]. [cited 2024 Apr 12]. Available from: https://github.com/gooofy/py-picotts
15. Evangelatos S. ttspico: Python simple text to speech module. [Internet]. [cited 2024 Apr 12]. Available from: http://github.com/sevangelatos/py-ttspico
16. SciPy - [Internet]. [cited 2024 Apr 12]. Available from: https://scipy.org/
17. Python Pillow [Internet]. [cited 2024 Apr 12]. Available from: https://python-pillow.org/

18. Clark JA. pillow: Python Imaging Library (Fork) [Internet]. [cited 2024 Apr 12]. Available from: https://python-pillow.org
19. MLflow | MLflow [Internet]. [cited 2024 Apr 12]. Available from: https://mlflow.org/
20. neptune.ai [Internet]. [cited 2024 Apr 12]. neptune.ai | The MLOps stack component for experiment tracking. Available from: https://neptune.ai/
21. Google Lens - Search What You See [Internet]. [cited 2024 Apr 8]. Available from: https://lens.google/
22. CamFind - Free Visual Search Engine for the Physical World [Internet]. [cited 2024 Apr 8]. Available from: https://camfindapp.com/
23. TapTapSee - Blind and Visually Impaired Assistive Technology - powered by CloudSight.ai Image Recognition API [Internet]. [cited 2023 May 2]. Available from: https://taptapseeapp.com/
24. LeafSnap - Plant Identification [Internet]. [cited 2024 Apr 8]. Leafsnap - Plant Identifier App, Top Mobile App for Plant Identification. Available from: https://leafsnap.app
25. Calorie Mama Food AI - Food Image Recognition and Calorie Counter using Deep Learning [Internet]. [cited 2024 Apr 8]. Available from: https://www.caloriemama.ai/
26. Narrator's Voice - TTS - Apps on Google Play [Internet]. [cited 2024 Apr 8]. Available from: https://play.google.com/store/apps/details?id=br.com.escolhatecnologia.vozdonarrador&hl=en
27. @Voice Aloud Reader (TTS) - Apps on Google Play [Internet]. [cited 2024 Apr 8]. Available from: https://play.google.com/store/apps/details?id=com.hyperionics.avar&hl=en
28. AI Voices - NaturalReader Home [Internet]. [cited 2024 Apr 8]. Available from: https://www.naturalreaders.com/
29. AI Voice Generator, Text To Speech, #1 Best AI Voice [Internet]. 2023 [cited 2024 Apr 8]. Available from: https://speechify.com/
30. T2S [Internet]. [cited 2024 Apr 8]. T2S. Available from: https://app-t2s.web.app/
31. iNaturalist [Internet]. [cited 2024 Apr 12]. iNaturalist. Available from: https://www.inaturalist.org/
32. Flora Incognita | EN – The Flora Incognita app – Interactive plant species identification [Internet]. [cited 2024 Apr 12]. Available from: https://floraincognita.com/
33. Plantum | Identify 10K+ plants, flowers and trees with up to 95% accuracy! Become an expert in plant diseases with Plantum! [Internet]. [cited 2024 Apr 12]. Available from: https://myplantum.com/
34. Pl@ntNet [Internet]. [cited 2024 Apr 12]. PlantNet Home. Available from: https://plantnet.org/en/
35. PlantSnap - Plant Identifier App, #1 Mobile App for Plant Identification [Internet]. [cited 2024 Apr 12]. Available from: https://www.plantsnap.com/
36. PictureThis [Internet]. [cited 2024 Apr 12]. PictureThis - Plant Identifier App | Plant Identification Online. Available from: https://www.picturethisai.com/
37. FlowerChecker, plant identification app [Internet]. [cited 2024 Apr 12]. Available from: https://www.flowerchecker.com/
38. Blossom - Plant Care Companion [Internet]. [cited 2024 Apr 12]. Available from: https://blossomplant.com/
39. Answers G. Garden Answers (Free Plant Identification) [Internet]. Garden Answers. [cited 2024 Apr 12]. Available from: https://www.gardenanswers.com/
40. What's that flower? [Internet]. [cited 2024 Apr 12]. Available from: https://whatsthatflower.com/

41. Download.com [Internet]. [cited 2024 Apr 12]. Garden Compass- Gardening & Plant Identification. Available from: https://download.cnet.com/garden-compass-gardening-plant-identification/3000-31713_4-76611415.html

42. GmbH P. Plantix. [cited 2024 Apr 12]. Plantix | #1 FREE app for crop diagnosis and treatments. Available from: https://plantix.net/en/

43. Plant care app - Keep your plants alive | Planta [Internet]. [cited 2024 Apr 12]. Available from: https://getplanta.com/

44. Foodvisor [Internet]. [cited 2024 Apr 12]. Foodvisor App - Nutrition Coaching - Eat Healthy & Lose Weight. Available from: https://foodvisor.io/

45. SnapCalorie - Single Photo Calorie Tracking [Internet]. [cited 2024 Apr 12]. Available from: https://www.snapcalorie.com/

46. LogMeal Food AI - Image API and Restaurant Checkout solutions. Food Detection and Food Tracking based on the most Advanced Deep Learning. [Internet]. [cited 2024 Apr 12]. Available from: https://www.logmeal.es/

47. App Store [Internet]. 2020 [cited 2024 Apr 12]. Yummy - Food Recognition. Available from: https://apps.apple.com/fi/app/yummy-food-recognition/id1532742213

48. Snap It™ - Lose It! [Internet]. [cited 2024 Apr 12]. Available from: https://www.loseit.com/snapit/

49. Samad S, Ahmed F, Naher S, Kabir MA, Das A, Amin S, et al. Smartphone apps for tracking food consumption and recommendations: Evaluating artificial intelligence-based functionalities, features and quality of current apps. Intelligent Systems with Applications. 2022 Sep 1;15:200103.

50. Wang CY, Bochkovskiy A, Liao HYM. YOLOv7: Trainable bag-of-freebies sets new state-of-the-art for real-time object detectors [Internet]. arXiv; 2022 [cited 2024 Feb 29]. Available from: http://arxiv.org/abs/2207.02696

51. GitHub - ultralytics/ultralytics: NEW - YOLOv8 ⍰ in PyTorch > ONNX > OpenVINO > CoreML > TFLite [Internet]. [cited 2024 Feb 29]. Available from: https://github.com/ultralytics/ultralytics

52. Redmon J, Divvala S, Girshick R, Farhadi A. You Only Look Once: Unified, Real-Time Object Detection [Internet]. arXiv; 2016 [cited 2024 Feb 29]. Available from: http://arxiv.org/abs/1506.02640
